# Superplasticizer Addition to Carbon Fly Ash Geopolymers Activated at Room Temperature

**DOI:** 10.3390/ma9070586

**Published:** 2016-07-18

**Authors:** Lorenza Carabba, Stefania Manzi, Maria Chiara Bignozzi

**Affiliations:** Department of Civil, Chemical, Environmental and Materials Engineering, University of Bologna, via Terracini, 28, Bologna 40131, Italy; stefania.manzi4@unibo.it (S.M.); maria.bignozzi@unibo.it (M.C.B.)

**Keywords:** geopolymer, admixtures, superplasticizers, workability, mechanical properties, porosity

## Abstract

Present concerns about global warming due to the greenhouse emissions in the atmosphere have pushed the cement industry to research alternatives to ordinary Portland cement (OPC). Geopolymer binder may constitute a possible breakthrough in the development of sustainable materials: understanding the effectiveness and the influences of superplasticizers on geopolymer systems is one of the essential requirements for its large-scale implementation. This study aims to investigate the possibility of using commercially available chemical admixtures designed for OPC concrete, to improve fresh properties of fly ash-based geopolymers and mortars. A special emphasis is laid upon evaluating their influence on mechanical and microstructural characteristics of the hardened material realized under room-temperature curing conditions. Results indicate that the addition of a polycarboxylic ether-based superplasticizer, in the amount of 1.0 wt. % by mass of fly ash, promotes an improvement in workability without compromising the final strength of the hardened material. Moreover, the addition of the polycarboxylic ether- and acrylic-based superplasticizers induces a refinement in the pore structure of hardened mortar leading to a longer water saturation time.

## 1. Introduction

Superplaticizers (SP) are widely used in concrete technology. These admixtures can be used as water reducers, maintaining a fixed workability or as plasticizers increasing workability without modifying the mix design. The first applications of chemical admixtures as water reducers date back to the 1940s with the adoption of lignosulfonate (LS), followed by the development in the 1960s of a high water-reducer based on sulphonated naphthalene formaldehyde (SNF) and sulphonated melamine formaldehyde (SMF). Finally, in the early 1980s, a new generation of superplasticizers based on polyacrylate polymers was designed [1]. Several studies [2,3,4,5] have been conducted on the chemistry and the operating principle of superplasticizers in cement matrix, confirming that their use allows enhanced mechanical and microstructural properties and durability performances of concrete. Therefore, the adoption of SP can be considered one of the most important improvements in concrete technology that contributed to its worldwide diffusion [1,6]. However, present concerns about global warming due to the accumulation of greenhouse gases in the atmosphere have pushed the cement industry to research alternatives to ordinary Portland cement (OPC). Indeed, the cement industry accounts for approximately 8% of global anthropogenic CO_2_ emissions considering that the production of 1 ton of cement releases an estimated 0.73–0.99 ton of carbon dioxide [7].

In this framework, geopolymer binder constitutes a possible breakthrough in the emerging technologies. Geopolymers are synthetized through the alkaline activation of low-calcium aluminosilicate materials and are mainly characterized by three-dimensional structure gels (N–A–S–H) [8]. Previous studies showed that these materials can achieve a minor greenhouse effect when compared to traditional cement [9,10,11]. However, when detailed analysis on geopolymer concrete are performed, taking into account several parameters, such as concrete mixture compositions, proximity, and availability of raw materials, energy/fuel types, and manufacturing process for the alkali activators, the reduction of estimated greenhouse gas emissions of geopolymer concrete varies in the range of 44%–64% [12], up approximately only 9% [13] when compared to OPC concrete. The sustainability of geopolymer can be implemented by using recycled waste powder (e.g., fly ash) instead of metakaolin as precursor [14], thus avoiding the high temperature calcination step. In addition, geopolymer concrete made from fly ash generally requires a lesser quantity of alkaline solution for the activation and, for this reason, it has a lower environmental impact than geopolymer concrete made from pure metakaolin [15]. Finally, the adoption of room temperature curing can reduce the CO_2_ emissions of geopolymer concrete by about 12.4% [13].

A large number of studies have confirmed the great potential of geopolymer systems as a construction material [16,17]. The majority of the research involves high-temperature curing and results show that, with a tailored mix design, a final product can be obtained with similar or even better properties than OPC-based material in terms of both mechanical and durability performance [18,19]. Moreover, geopolymer showed promising results as high-temperature-resistant products [20,21] and lightweight materials [22,23]. On the contrary, only few studies [24,25] focus on performances of geopolymers cured at room temperature which are more energy saving and cost effective.

As happened for OPC concrete, understanding the effectiveness and influence of superplasticizers in geopolymer systems is one of the essential requirements for its large-scale acceptance as a building material. However, only few studies have been conducted on this topic and results are often contradictory on establishing which chemical structure of the admixtures can promote a significant plasticizing effect [26,27,28,29,30,31]. In addition, although it is well known that, in the case of traditional OPC concrete, the use of SP can increase the air-entrainment during the mix or can affect porosity and pore size distribution of the hardened material [32,33], to the best of our knowledge published studies dealing with effects of superplasticizers on porosity of fly ash-based geopolymer are missing. For this reason, research focused on this topic is necessary as porosity is strictly connected to the durability issues of final products.

This paper aims to further investigate the influence of commercially available superplasticizers designed for OPC concrete on properties of geopolymers and geopolymer mortars. Geopolymers here investigated were obtained through room-temperature alkali activation of carbon fly ash with sodium hydroxide and sodium silicate solutions. Several types of chemical admixtures, which vary for the chemical structure, were added during the mixing process. Fresh and hardened properties of the final products were analyzed with a special focus on porosity and its distribution in order to get a more comprehensive overview of the influence of superplasticizers in geopolymer materials.

## 2. Materials and Methods

### 2.1. Raw Materials

Low-calcium coal fly ash (class F FA) was sourced from Enel Produzione S.p.A. UB Torrevaldaliga Nord power station located in Italy (Civitavecchia, Rome, Italy) and was supplied by General Admixtures S.p.A. (Ponzano Veneto, Treviso, Italy). The fly ash complies with the EN 450-1 European standard [34] and is approved for use in the cement and concrete industry. The chemical composition of FA is given in Table 1 [24]. The fly ash exhibits a *d*_50_ = 17.5 μm, its mineralogical composition includes crystalline phases of quartz, mullite, and maghemite, and an amorphous content equal to 69 ± 0.8 wt. % [24]. A detailed study on the structural characteristics of the fly ash used as precursor is reported elsewhere [35].

Sodium silicate and sodium hydroxide (8 M NaOH) were used as activating solutions. The sodium silicate solution was supplied from Ingessil S.r.l (Verona, Italy) with a trade name Reoflux B and with the following chemical composition: 29.86% SiO_2_, 14.43% Na_2_O, 55.71% H_2_O, and SiO_2_/Na_2_O ratio equal to 2.07.

For the preparation of the geopolymer mortars, a normalized silica sand with a fixed grain size distribution (*d*_max_ = 2 mm) was used as the fine aggregate. The sand complies with the EN 196-1 standard [36].

Finally, seven types of commercially available superplasticizers, commonly used in the OPC concrete industry, were used. Their chemical structure and main physical characteristics are reported in Table 2 together with their identification label. The SP were added in the mixture as they are supplied, with the only exception of LGa and LGb, which were diluted with water before use, according to the producer’s datasheet for OPC concrete application. The SP content is always expressed in geopolymer mixtures as a weight percent by mass of fly ash.

### 2.2. Geopolymer Preparation

Geopolymer was prepared by mixing, for four minutes, fly ash (65.7 wt. %) with alkaline solutions (24.7 wt. % of sodium silicate and 5.0 wt. % 8M NaOH) and water (4.6 wt. %). The mix design was adjusted according to previous results [37] in order to achieve a molar ratio of Na_2_O/SiO_2_ equal to 0.12, determined considering the total content of sodium oxide and silica present in the sample. The admixture, when presents, was added at the end of the mixing process. Two different contents of admixture by mass of FA (0.6 and 1.0 wt. % by mass of FA) were selected with the aim to investigate its influence on the final product. Geopolymers were labelled according to the chemical structure of the admixture, followed by its amount expressed in percentage by mass of precursor (i.e., PCE_06 refers to a sample with 0.6 wt. % of polycarboxylic ether by mass of fly ash). Furthermore, a geopolymer mixture without superplasticizer was prepared as a reference sample and named GP.

### 2.3. Geopolymer Mortar Preparation

Mortar samples were prepared with the same molar ratio of Na_2_O/SiO_2_ previously used for geopolymers. SMF, PCE, ACRa, and ACRb were tested as superplasticizers in the amount of 0.6 wt. %, with the only exception of PCE, which was also tested in the amount of 1.0 wt. %. A geopolymer mortar without SP was realized as a reference. Mixtures were prepared in a Hobart mixer introducing fly ash (23.8 wt. %), 8M NaOH (1.8 wt. %) and sodium silicate (9.0 wt. %) solutions. Mixing at low speed was operated for 30 s, after which sand (64.3 wt. %) was gradually added during the following 30 s. Afterward, water (1.1 wt. %) was introduced, followed by the superplasticizer, when present. The mixing was paused for 90 s and resumed for an additional 60 s at high speed. At this stage, tests on the fresh mortar were carried out and, finally, the slurry was poured into molds and mechanically vibrated in order to obtain prismatic samples (40 × 40 × 160 mm^3^). The molds were stored closed in plastic bags at room temperature (*T* = 23 ± 2 °C) for 24 h after which samples were de-molded and cured under the same conditions for a further 27 days. Setting time occurred regularly when the different SP were added and no delay was registered in the de-molding procedure. Mortar samples were labelled according the aforementioned legend used for geopolymer, where the acronym highlights the chemical structure and the amount of the superplasticizer.

### 2.4. Testing Methods of Fresh Properties

Workability of geopolymer was evaluated through the minislump test [38]. After mixing, geopolymer mixture was poured into a truncated conical mold (*d*_min_ = 19 mm, *d*_max_ = 38 mm, *h* = 57 mm). The mold was lifted up and the mean diameter of spread paste was measured after one minute. This test was performed immediately after mixing (*t* = 0’) and repeated after 5, 15, and 30 min on the same mixture, in order to evaluate the variations of workability during time.

Workability of the geopolymer mortars was measured by means of the conventional flow table test, in accordance with the EN 1015-3 [39]. The workability, expressed in terms of consistency %, was determined as the percentage of the difference between the average diameter of the spread mixture and the diameter of the conical ring (100 mm) divided by the diameter of the conical ring (100 mm).

In order to evaluate the influence of SP on the air entrainment in the fresh mixture, the air content of the fresh geopolymer mortars was determined according to EN 1015-7 [40].

### 2.5. Testing Methods of Hardened Properties

The mechanical characterization of geopolymer mortars was carried out by performing compressive and flexural strength test according to EN 196-1 [36] on mortar specimens (40 × 40 × 160 mm^3^) cured for 28 days in sealed plastic bags at room temperature. Flexural and compressive strengths were determined by an Amsler–Wolpert machine (maximum load: 100 kN, Ludwigshafen, Germany) at a constant displacement rate of 50 mm/min. The reported flexural and compressive strengths are the average values determined on the basis of three measurements. Moreover, an evaluation of ultrasonic pulse velocity was performed by using a commercial ultrasonic testing instrument (Matest, Bergamo, Italy) made up of a pulse generator and two transducers (55 kHz) that were positioned at opposite ends of 160 mm long sample. The reported ultrasonic pulse velocity is the average value determined on the basis of three measurements. Finally, the bulk density was calculated as dry mass divided by the sample volume.

For the investigation of the pore structure of geopolymer mortars, low magnification imaging was performed using the Olympus SZX10 optical microscope (Olympus, Tokyo, Japan). The optical microscopy analysis was used in combination with the image analysis software LAS V3.8 (Leica, Wetzlar, Germany) for obtaining information on macroporosity of the hardened specimens. In addition, after 28 days of curing the pore size distribution of samples (about 1 cm^3^) was investigated by mercury intrusion porosimetry (MIP) (Carlo Erba 2000) equipped with a macropore unit (Model 120, Fisons Instruments, Milan, Italy). This technique is based on the intrusion of a non-wetting fluid (mercury) into the pore structure under increasing pressure. The Washburn equation [41] is used to relate the pressure to pore size. A mercury surface tension of 0.48 N/m and a contact angle of 141.3° were set for the MIP measurement.

Finally, a capillary water absorption test was performed according to EN 15801 [42], in order to evaluate the influence of SP on the interconnection of the pores of the hardened mortars. The test was conducted on six cylindrical samples (*d* = 20 mm, *h* = 40 mm) for each type of mortar, cored from a bigger specimen (40 × 40 × 160 mm^3^). As required by the standard, the amount of water absorbed per unit area at the time *t*_i_ is expressed by the capillary water absorption curve.

### 2.6. Admixtures Characterization

Fourier transform infrared spectroscopy (FT-IR) measurements were carried out by means of a PerkinElmer Spectrum Two instrument (Fremont, CA, USA). The spectra were recorded by attenuated total reflection (ATR) sampling technique on samples of PCE and ACRa admixtures, previously dried in a vacuum dryer. In order to test the chemical stability of PCE and ACRa in the alkaline solution media, the admixtures were also mixed with an 8 M NaOH solution (weight ratio 1:1). After 30 min of stirring, the solution was dried in a vacuum dryer and the sample spectra was registered.

## 3. Results

### 3.1. Workability

**Figure 1:**
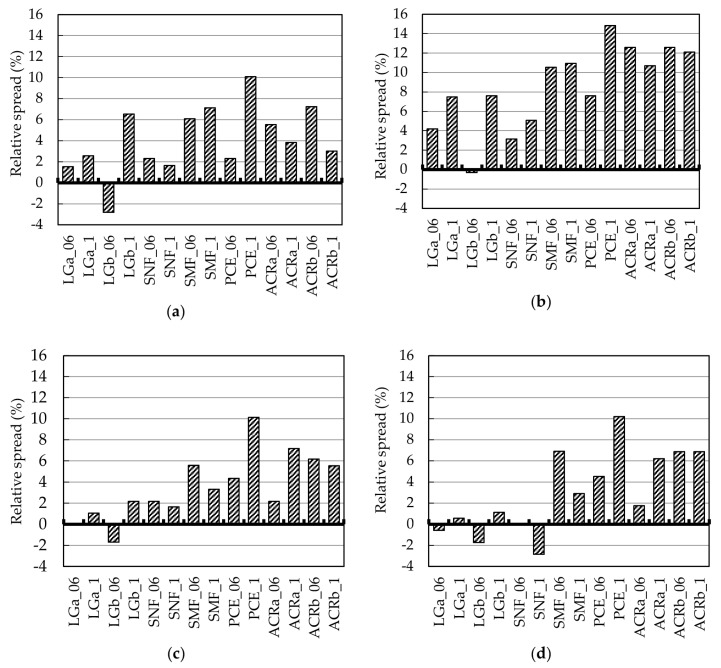
Workability of geopolymer expressed as relative spread (increase or decrease in %) of the geopolymer mixtures immediately (**a**) after mixing; (**b**) after 5 min; (**c**) 15 min; and (**d**) 30 min.

A workability test on fly ash-based geopolymer was carried out in order to evaluate the effectiveness of the investigated SP and it has been expressed as consistency for mortar samples and relative spread for geopolymers. The relative spread is defined as the difference, expressed in percentage, between the spread of the tested mixtures containing SP and the spread of GP mixture divided by GP spread diameter. Figure 1 reports the results of the minislump test as function of time (0, 5, 15, 30 min after mixing). Lignosulphonate- and naphthalene-based admixtures (LGa, LGb, SNF) do not significantly improve workability of geopolymers either in the case of the added amount of 0.6 wt. % or when 1.0 wt. % of SP is used. Moreover, in the cases of LGb_06 and SNF_1 samples, the results show a reduction in spread of the mixtures when compared to GP. On the contrary, geopolymers containing SMF, PCE, ACRa, and ACRb superplasticizers show a spread higher than GP during all the tested times (0, 5, 15, and 30 min). Increasing the amount of SP from 0.6 to 1.0 wt. % is effective only when PCE is used, whereas it does not always correspond to an increase in workability for SMF, ACRa, and ACRb.

At *t* = 0’, the relative spreads of SMF_06, PCE_1, ACRa_06, and ACRb_06 mixtures are equal to 6.1%, 10.1%, 5.5%, and 7.2%, respectively, thus highlighting an increase in the flow diameter of these mixtures up to 10.1% compared to GP. For this reason, these SP were also tested on geopolymer mortar specimens.

**Figure 2:**
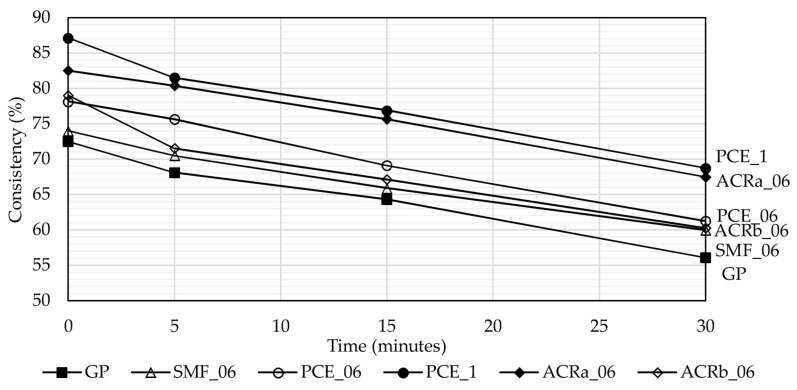
Consistency of geopolymer mortars as function of time (average of four measurements). Standard deviation (δ) is not reported in the plot for clarity’s sake however it is in the range of ±5.

Figure 2 shows the flow table test results obtained on mortar samples. Consistency is expressed as an average of four different tests per mixture. At *t* = 0’, PCE_06, PCE_1, ACRa_06, and ACRb_06 show a consistency in the range of 74%–87%, which is higher than the reference sample one. ACRa_06 and PCE_1 mixtures exhibit the highest values and at *t* = 30’ they show consistency equal to 69% ± 2% and 68% ± 4%, respectively, thus exhibiting a workability very close to the one of GP at *t* = 0’ (73% ± 4%).

As a general trend, mortar workability decreases with time with a similar rate for all the investigated mixtures and, for PCE, higher values are always registered when its content is 1 wt. % than 0.6 wt. %.

### 3.2. Air Content

The determination of air content is essential for understanding if the detected workability improvement is ascribed to an increase of air entrainment due to the use of SP rather than to their plasticizing effect [43]. The air content test was conducted on the reference mixture (GP) and on PCE_1 and ACRa_06, the two mixtures showing the best results on the flow table test.

The consistency of mortars immediately after mixing (*t* = 0’) and the relevant air content % are reported in Table 3. From the results, no correlation between the workability improvement and air content is found. Indeed, PCE_1 mix exhibits an equal or lower air content than the other tested mixtures, even if it has the highest consistency.

### 3.3. Superplasticizer Chemical Stability

**Figure 3:**
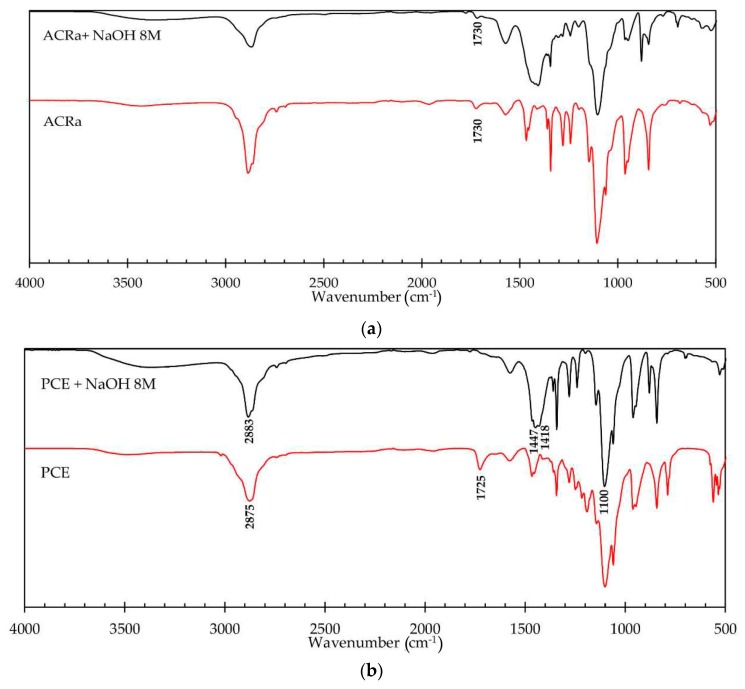
FT-IR spectra for (**a**) ACRa; and (**b**) PCE superplasticizers dried in a vacuum dryer and after mixing with an 8 M NaOH solution.

The infrared spectra for PCE and ACRa dried in a vacuum dryer and after mixing with an 8 M NaOH solution are reported in Figure 3. Infrared spectroscopy is a technique largely used for studying superplasticers in cement and alkali activated materials [30,44,45,46]. For both the superplasticizers the band at 3450–3400 cm^−1^ corresponding to OH^−^ group slightly increase comparing the spectrum of the dried admixture with the one where the admixture was treated with NaOH solution, thus indicating a good chemical stability of PCE and ACRa in the alkaline medium. Indeed, this peak usually strongly increases when polymer degradation occurs according to the increase content of the OH terminal chain group formed during hydrolysis. Moreover, for ACRa spectra (Figure 3a) the band at 1730 cm^−1^ corresponding to C=O carboxylic derivatives, remains almost unchanged when the alkaline treatment is carried out confirming a good chemical stability. Conversely, for PCE (Figure 3b) the band at 1725 cm^−1^ practically disappears, but only one (at 1418 cm^−1^) of the two characteristic bands (1575 and 1418 cm^−1^) corresponding to carboxylate groups appears as a shoulder of the peak at 1447 cm^−1^. Thus, for PCE, a certain degree of main chain degradation can be hypothesized, however a large part of the lateral chains comprising ether bond (peak at 1100 cm^−1^) still remains bound and able to exert its action of steric hindrance [30].

### 3.4. Mechanical Properties

**Figure 4:**
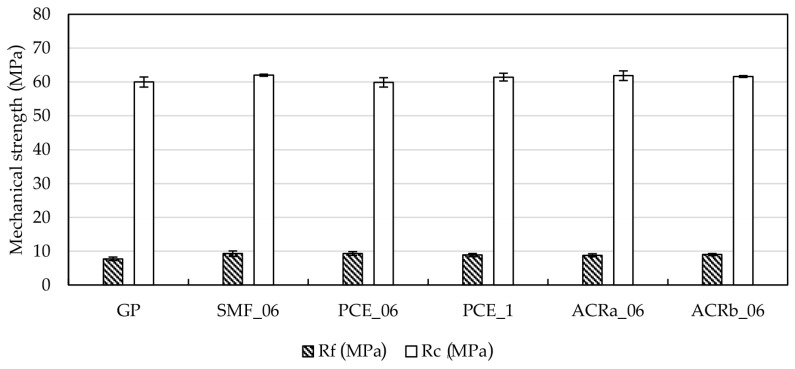
Mechanical properties of hardened geopolymer mortars after 28 days of curing at room temperature (values are reported as average of three measurements).

Flexural (Rf) and compressive (Rc) strengths were evaluated to determine if the use of SP affects the mechanical properties of the hardened materials. The results are presented in Figure 4: the reference sample GP shows a compressive strength equal to 60.0 ± 1.5 MPa and the relevant values for the samples containing SP are between 60–62 MPa. A similar behavior is also detected for flexural strengths, where GP exhibits a value of 7.7 ± 1.1 MPa and samples with SP are about 9 MPa.

All of the ultrasonic pulse velocity measurements are in the range of 3.4–3.7 km/s. During the test, the ultrasonic pulse can be reflected or refracted if discontinuities, voids or cracks are present in the sample. Therefore, all the samples appear quite similar, indicating that the addition of SP does not affect substantially the final product. This result is confirmed by the bulk density which is 2.1–2.2 g/cm^3^ for all the investigated specimens.

The mechanical behavior highlights that the presence of SP does not interfere with the regular development of compressive and flexural strengths, thus highlighting that the action of SP is limited to increase the workability as expected. Such a behavior is particularly evident when PCE is added, where to an increase in its content corresponds a higher workability and very similar mechanical performances.

### 3.5. Microstructural Characterization

In order to better elucidate the effect of SP on samples microstructure, optical microscopy analysis and mercury intrusion porosimetry were carried out on the reference mix GP and the best performing mixtures PCE_1 and ACRa_06, after 28 days of curing.

**Figure 5:**
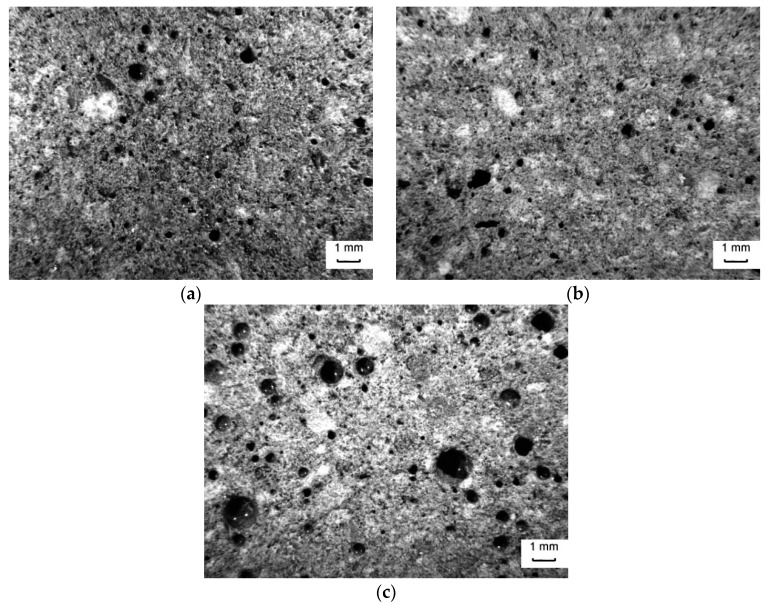
Optical images of geopolymer mortars after 28 days of curing: (**a**) PCE_1; (**b**) ACRa_06; and (**c**) GP. (Magnification 8×).

The images reported in Figure 5 exhibit a different amount of total porosity. GP sample shows a large number of spherical pores of diameter up to 1.57 mm, whereas PCE_1 and ACRa_06 show pores with a maximum diameter of 0.87 and 1.07 mm, respectively.

**Figure 6:**
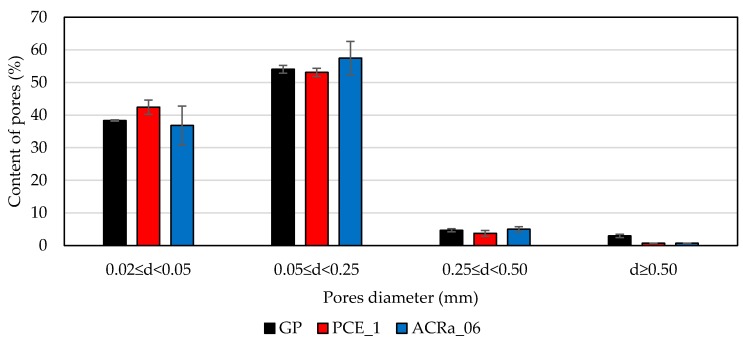
Pore distribution obtained via image analysis of two sections of hardened mortar (investigated area = 145 mm^2^).

Reporting the content % of pores arranged per ranges of pore diameters (d) obtained by imaging analysis of two analyzed areas per sample (Figure 6), it is confirmed that GP is the sample with the highest amount of pores with *d* ≥ 0.50 mm. The content of pores with diameters in the range 0.05 ≤ *d* < 0.25 mm and 0.02 ≤ *d* < 0.05 mm is similar for all the formulations. The total porosity (*P*_t_) determined by imaging analysis follow this order *P*_t__GP (11.7%) > *P*_t__ACRa_06 (9.0%) > *P*_t__PCE_1 (6.8%) indicating that even if the air content in the mortar samples at the fresh state is similar, the lowest workability of GP does not allow the entrapped air to easily quit, thus promoting large pore formation.

**Figure 7:**
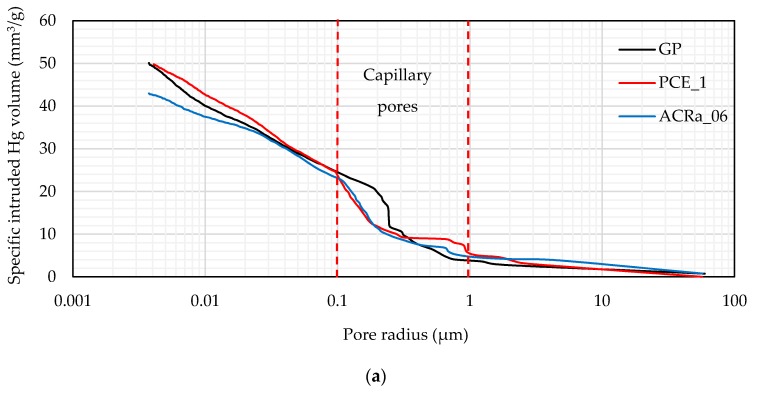

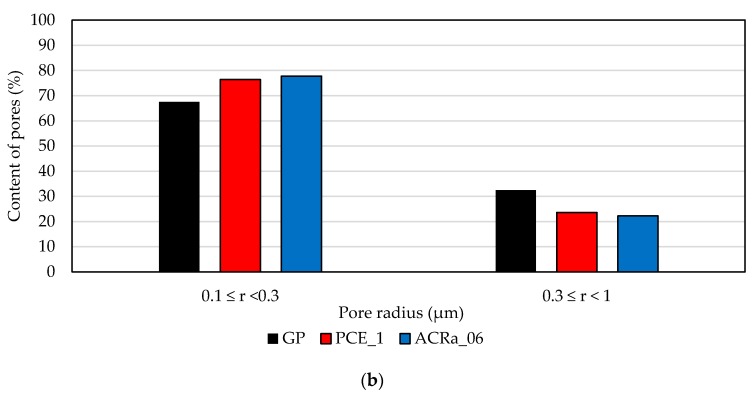
(**a**) Pore size distributions curves of the investigated geopolymer mortar samples; (**b**) content % of pores arranged per ranges of pore radius (*r*) within the range of 0.1 ≤ *r* < 1 μm.

The results of MIP analysis are plotted in Figure 7. Unlike microscopy analysis, MIP allows the investigation of open porosity between 0.04–70 μm. The open pore size distribution curves of samples GP, PCE_1, and ACRa_06 appear to have similar trend, even if the main differences occur in the pore radius range 0.1–1 μm, the so-called capillary pores. In particular, the detected average pore radius is 0.21, 0.12, and 0.16 μm for GP, PCE_1, and ACRa_06, respectively, highlighting a pore refinement for samples where SP was added. Reporting the pores content at two different intervals of radius in the capillary range, it is highlighted that GP mortar contains a lower and higher content of pores with radius between 0.1 ≤ *r* < 0.3 and 0.3 ≤ *r* <1 μm, respectively, than PCE_1 and ACRa_06 mortar samples, whereas the total content of capillary porosity is very similar for all the three mixtures (about 40% of the relevant total open porosity).

**Figure 8:**
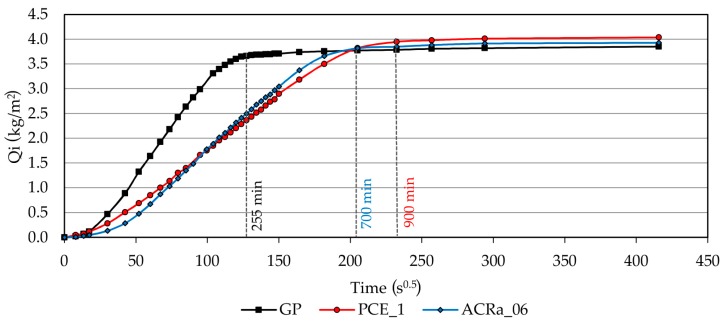
Capillary water absorption test results for GP, PCE_1, and ACRa_06 samples. The time of saturation for each sample is indicated with a vertical line.

In order to investigate the degree of pores interconnectivity, a capillary water absorption test was also carried out. The data (Figure 8) shows that all the three mixtures, at the end of the test, reach similar values of water absorbed per unit area, thus indicating a similar total open porosity interconnectivity. However, GP sample saturates after 225 min (≈4 h), whereas ACRa_06 and PCEa_1 samples reach the saturation after 700 min (≈12 h) and 900 min (15 h), respectively. The results show that a different distribution in capillary pores as detected by MIP plays an important role in the saturation rate. The highest content of small capillary pores (0.1 ≤ *r* < 0.3 μm) detected for PCE_01 and ACRa_06 samples slows down the saturation rate [47] and, consequently, increases the material durability.

## 4. Discussion

The superplasticizers currently available for traditional OPC concrete are not designed to work with geopolymer systems. Despite the fact that, in terms of performance, geopolymer and OPC-based materials can be comparable, it has to be highlighted that the reaction mechanism and products of the two systems are completely different, as reported in the literature [48,49]. However, in the absence of specific superplasticizers designed for alkali-activated systems, it is useful to understand if the available SP can be successfully adopted for the production of geopolymers.

In the presented study, superplasticizers have been used without modifying the original mix design, with the aim to improve the workability of the fresh slurry and to control if their addition can affect other important properties of the hardened material, such as mechanical strength and porosity. Results concerning the consistency of the geopolymers and mortars show that the latest generation admixtures (PCE- and acrylic-based types) are more effective in terms of workability improvement than lignosulphonate-, naphthalene-, and melamine-based SP. Such differences can be ascribed to the different chemical structure. Indeed, LS-, SNF-, and SMF-based superplasticizers rely on an electrostatic repulsion: in the cement based materials, the electrostatic attractive forces among cement particles, which generate agglomeration, are neutralized by the adsorption of anionic polymers negatively charged for the presence of the SO_3_^−^ groups on the surface of cement particles. The dispersion is obtained by the electrostatic repulsion produced by the negatively charged SO_3_^−^ groups on the opposite side of the main polymer chain [50]. Regarding the latest generation of SP, the dispersion mechanism is more related to a steric hindrance effect generated by the side chains of the polymer, than to the presence of negatively-charged anionic COO^−^ group, which is responsible for the adsorption of the polymers on the surface of cement particles [50].

Although a study [30] related to the efficiency of SP in alkali-activated slag systems revealed that the majority of SP used for OPC-based binder seems to degrade in the high alkaline environment, the chemical structure of PCE-based superplasticizer and the presence on numerous lateral side chains can prevent the tendency of binder particles to agglomerate [27]. Moreover, a recent study [51] on the adoption of PCE-based superplasticizer in alkali-activated slag pastes confirms that by acting on the molecular architecture of the PCE, its performance as a superplasticizer is improved. In this paper it has been demonstrated that the investigated PCE and ACRa superplasticizers are stable in the alkaline medium and, even when some degradation occurs (for PCE), the steric hindrance due to the side chains is still effective to break fly ash particle agglomeration, thus avoiding workability loss.

The results here presented also show that the use of ACRa and PCE up to 1 wt. % does not influence the compressive and flexural strengths of the hardened fly ash-based geopolymer mortars. Therefore, their addition does not disturb the geopolymerization process and promotes the formation of products with performance comparable with those usually determined for a high-strength class cement (e.g., 52.5 MPa). Microstructure analysis highlights that the addition of PCE or ACRa promotes a lower total porosity, decreasing the contribution of close pores usually formed by entrapped air. The MIP results show that all the three mixtures have a comparable pore size distribution typical of fly ash geopolymer materials with a 40% of pores in the capillary range (0.1–1 μm) [52]. The class of pore from few microns up to 10 μm in diameter depends on the retreat of the surface of dissolving fly ash grains after gelation [53]. The addition of PCE_1 and ACRa_06 slightly decreases the average pore radius, favoring a pore refinement, and increases the pore content with dimensions in the range 0.1–0.3 μm. Accordingly, both of these issues lead to an increase of the saturation time for PCE_1 and ACRa_06 samples, thus suggesting a longer durability for these products [54,55,56].

## 5. Conclusions

In the absence of specifically-designed admixtures for geopolymer materials, understanding if cement-designed superplasticizers can be useful for alkali-activated systems is a topic of great importance. The results here discussed allow drawing the following conclusions:

- modified acrylic and polycarboxylic ether-based superplasticizers show the highest efficacy in improving the workability performances of carbon fly ash geopolymer mixtures. In particular, the best results are obtained by using a PCE-based superplasticizer in the amount of 1.0 wt. % by mass of the solid precursor;
- both these investigated superplasticizers, belonging to the last generation of admixtures, provide a satisfying workability improvement in the geopolymer mixtures without increasing the air content at the fresh state and affecting the mechanical properties developed during room temperature curing;
- modified acrylic and polycarboxylic ether-based superplasticizers allow obtaining geopolymer mortars with a more compact microstructure thanks to the improvement in workability which facilitates the entrapped air evacuation and to the refinement of the average pore radius. As a consequence, capillary test shows a slower water saturation time than the reference mortar one in which no superplasticizer was added.

Finally, the use of superplasticizers can be a very effective tool in promoting the practical use of carbon fly ash geopolymers in civil engineering where the need to work with low environmental impact materials is becoming an urgent matter according to international directives. The building sector will benefit from geopolymers as soon as their use is consolidated in all of their technical aspects.

## Figures and Tables

**Table 1:** Chemical composition of fly ash.

Chemical Compositions (wt. %)	
SiO_2_	49.37
Al_2_O_3_	29.23
Fe_2_O_3_	2.71
CaO	6.63
MgO	1.05
SO_3_	0.33
Na_2_O	<0.05
K_2_O	0.60
LOI	3.28

**Table 2:** Chemical and physical properties of the investigated superplasticizers.

Label	Chemical Structure	Solid Content (%)	Appearance Color	Density (g/cm^3^)
LGa	Lignosulphonate	48	Brown liquid	1.20
LGb	Sodium lignosulphonate	50	Brown liquid	1.27
SNF	Polynaphthalenmethan sulphonate	100	White powder	0.55
SMF	Sulphonated melamine	100	Brown powder	0.80
PCE	Polycarboxylic ether	17	Brown liquid	1.04
ACRa	Modified acrylic	31	Yellow liquid	1.09
ACRb	Acrylic acid copolymer	21	Yellow liquid	1.08

**Table 3:** Consistency and air content of GP, PCE_1, and ACRa_06 (average values of four and two measurements, respectively).

Mixture	Consistency (%)	Air Content (%)
GP	73 ± 4	4.9 ± 0.5
PCE_1	87 ± 3	4.9 ± 0.1
ACRa_06	83 ± 5	5.1 ± 0.1
